# Navigating virtual selves: validation of the German version of the presentation of online self scale

**DOI:** 10.3389/fpsyg.2024.1435691

**Published:** 2024-09-11

**Authors:** Lynne Marie Stöven, Philipp Yorck Herzberg, Fabio Ibrahim

**Affiliations:** Department of Personality Psychology and Psychological Assessment, Helmut-Schmidt-University, Hamburg, Germany

**Keywords:** online self-presentation, social media behavior, online self-presentation assessment, POSS German version, Instagram followers, online identity, authentic self

## Abstract

The Presentation of Online Self Scale for Adults (POSSA), originally developed by Strimbu et al. is a well-regarded instrument for assessing online self-presentation. This study evaluated the factorial structure, reliability, and validity of the German adaptation of POSSA. A CFA analysis confirmed a satisfactory fit for the proposed three-factor model, as evidenced by a CFI of 0.919, a TLI of 0.902 and a RSMEA of 0.075. The subscales of the German POSSA demonstrated high internal consistency. Additionally, convergent validity was established through significant correlations with the Impostor-Profile 30 (IPP), affirming the interpretive accuracy of the subscale scores. Specifically, the Adaptable Self and Freedom of Self Online subscales positively correlated with IPP measures of Alienation and Other-Self-Divergence, whereas the Authentic Self subscale inversely correlated with these measures. Moreover, the German POSSA scores accounted for variance in the number of Instagram followers, surpassing the predictive power of self-esteem alone. Notably, the Adaptable Self factor was positively associated with the follower count, while the Freedom of Self Online factor displayed a negative association. Collectively, these findings underscore the DE-POSSA as a robust tool for assessing self-presentation behaviors in German-speaking populations and highlight its potential for cross-cultural research in online interpersonal interactions.

## 1 Introduction

Self-presentation is a multifaceted psychological phenomenon that encompasses the conscious and subconscious strategies individuals employ to shape the impressions they convey to others (Goffman, 1959; Schlenker, 1985). In the digital era, the proliferation of social media and other online platforms has intensified the complexities of self-presentation, providing individuals with unprecedented opportunities to curate and control their public personas.

Self-presentation is a psychological concept that refers to the process by which individuals control or manage the information they convey about themselves to others (Schlenker, 1985). It involves strategic efforts to shape others’ perceptions, opinions, and impressions, with the aim of presenting oneself in a particular light (Goffman, 1959). It further involves consciously or unconsciously managing and controlling the impressions others form about oneself. This concept delves into how people shape their behaviors, appearance, and communication to create specific impressions or to maintain a particular image. Self-presentation is a fundamental aspect of social behavior and occurs in various contexts, including face-to-face as well as online interactions (Hollenbaugh, 2021).

The exponential ascent of social networking platforms, which established as an obligatory tool in interpersonal dynamics, has provided novel avenues for self-presentation. Numerous functionalities as well as the varying degree of anonymity, these platforms afford users an expansive repertoire of self-expressive possibilities, virtually boundless in scope (Hollenbaugh, 2021; Nadkarni and Hofmann, 2012; Schlosser, 2020). Online, individuals often have more control over the information they present. They can curate their online profiles, choose what to share, and edit or delete content. This control allows for a more deliberate and strategic presentation compared to offline interactions, where immediate control over information is limited. Further, online environments sometimes offer a level of anonymity, leading to disinhibition - people may feel freer to express opinions or behaviors they might not display offline due to reduced social consequences. The transformative impact of social networking sites on self-presentation is undeniable. Variances in norms, anonymity, and information control underscore the distinct rules governing online self-expression compared to face-to-face interactions. The onus lies on the user to navigate these unique features, determining the extent to which online and face-to-face self-presentation intersect or diverge. Given the growing significance of this topic, Strimbu et al. (2021) have validated the Presentation of Online Self Scale in adults (POSSA) to assess online self-presentation behaviors. However, this instrument is currently only available in English. Considering the global importance of online self-presentation, the present study aims to validate a German version of the POSSA (DE-POSSA).

### 1.1 Online self-presentation

Many studies indicate that peoples online and offline personalities are rather consistent (Fullwood et al., 2016; Hollenbaugh, 2021; Marriott and Buchanan, 2014), while other research finds the opposite (Casale and Fioravanti, 2018; Michikyan et al., 2014; Twomey and O'Reilly, 2017). In this context, it is first necessary to differentiate between two terms that are often used generically - self-presentation and self-disclosure. Self-presentation and self-disclosure are both important aspects of online social interactions, but they differ in their focus and intention. Self-disclosure involves revealing personal information, thoughts, feelings, or experiences to others, regardless of the creation of a certain impression. On the other hand, self-presentation involves controlling information to shape others’ perceptions of oneself (Schlenker, 1985). In online contexts, self-presentation often includes elements such as profile pictures, bios, status updates, and the content shared or posted. Despite the lack of non-verbal cues, individuals can create carefully crafted online personas through selective self-disclosure, asynchronous communication, and the use of text-based cues to convey emotions and personality traits effectively (Walther, 2007). By leveraging these functionalities, users can carefully curate their online persona to highlight certain aspects of oneself, aiming to create a favorable impression. Thus, self-presentation is goal directed and does not always reflect the complete reality of a person’s life or personality (Schlenker, 1985).

Certain characteristics of social network sites (SNSs) and interaction via them can influence the extent of self-presentation. Trepte’s Social Media Privacy Model highlights the significant impact of these SNS-affordances (i.e., persistence, anonymity, editability, and association) on self-presentation (Trepte, 2021). Based on these affordances, activities such as profile customization, selective sharing, and audience segmentation allow individuals to control the visibility of their personal information and tailor their self-presentation to different social contexts. These tools enable users to strategically craft their online personas, balancing the desire for social connectivity with the need for privacy. Thus, users can navigate the complexities of self-presentation, maintaining a desired public image while safeguarding their private selves. Schlosser (2020) identified three influencing factors. Firstly, the asynchron*y* of communication plays a role in increasing self-presentation. While a high degree of spontaneity, unpredictability, and arbitrariness is inherent to synchrony in face-to-face interaction, online interaction offers a certain degree of asynchrony. It is possible to react immediately, but not necessary. This means that the reaction or interaction can be better considered and planned in order to convey the desired image. Goals and strategies for self-presentation on SNSs vary widely among users and are influenced by individual motives, social contexts, and the desired outcomes (Trepte and Reinecke, 2013). While many users strive to present themselves favorably to gain social approval and support, this is not a universal or constant aim. Some users might engage in negative self-presentation to elicit empathy, support, or comfort from others, leveraging the platform’s affordances to address personal struggles (Seidman, 2013; Utz, 2015). Secondly, multiple audiences are a typical characteristic of online interaction. The created content reaches a very heterogeneous group compared to face-to-face interactions. Such multiple audiences can include both real friends and purely virtual friends. Consequently, the characteristics and preferences of the members of ones’ online network may differ greatly from one another so that the audience is difficult to estimate. This has direct implications for authentic self-presentation. Authenticity involves being genuine and true to oneself, which may not always align with what is socially or professionally advantageous (Ellison et al., 2006). For example, sharing personal vulnerabilities may be authentic but not necessarily advantageous in a professional context. Conversely, presenting oneself in a highly curated and idealized manner may be advantageous in terms of social approval but lacks authenticity in a more familiar community (Goffman, 1959). Navigating these varied audience dynamics can present challenges for individuals seeking to manage their online image. They may feel pressure to reconcile potentially conflicting perceptions and expectations from different segments of their online social network. For example, individuals may present different aspects of themselves to different audiences in order to maintain consistency within each subgroup while still appealing to the diverse interests and preferences of their entire online network. This juggling act underscores the complex interplay between self-presentation, audience management, and social identity construction in online environments. Social identity construction refers to the process by which individuals form and develop their sense of self in relation to their social groups and environments (Ellemers and Haslam, 2012). This involves adopting and internalizing the norms, values, and behaviors associated with particular social categories. Social identity construction is influenced by social interactions and the feedback received from others. SNSs offer the possibility of audience feedback, e.g., through likes or comments. This leads to people sharing content for which they expect positive feedback. Due to the multiple audiences mentioned above, this can again become a difficult balancing act between authenticity and desirability in self-presentation.

The availability of immediate feedback mechanisms on social media platforms introduces a dynamic where individuals may feel compelled to tailor their self-presentation to garner favorable reactions from their audience (Rui and Stefanone, 2013). This desire for validation can influence the type of content shared and the manner in which it is presented, potentially leading to a distortion of one’s authentic self in favor of presenting a more socially desirable image (Zheng et al., 2020). In addition to anonymity, Hollenbaugh (2021) identified two other affordances that influence self-presentation, namely visibility (i.e., how easily published content is accessible online) and persistence, i.e., how long content remains available online which vary between different SNSs. High visibility can lead to increased self-awareness and cautious behavior, as users are cognizant of the broad and potentially unintended audience their content may reach. According to Trepte (2021), this results in a more controlled and polished self-presentation, as users strive to meet social norms and expectations to avoid negative judgment. Further, the persistence of online content can influence self-presentation by encouraging users to consider the long-term implications of their posts. They might opt for content that reflects positively on their identity over time, aware that past actions and expressions can resurface and impact future opportunities or relationships (Trepte, 2021). Trepte (2021) further highlights the role of anonymity which provides a sense of security and freedom by allowing users to interact without revealing their true identities. This enables users to experiment with different aspects of their identity and express themselves more openly, without the fear of real-world repercussions. Consequently, anonymity becomes a crucial affordance in social media that shapes users’ behavior and the dynamics of social privacy, influencing how they present themselves to others in these digital spaces.

In summary, the characteristics of SNSs, including the permanence of content, asynchronous communication, anonymity, and broad audience reach, present unique opportunities and challenges for online self-presentation that differ significantly from face-to-face interactions. Online self-presentation offers individuals greater control over their image, allowing for selective disclosure and the creation of idealized personas. This can be particularly advantageous for introverted individuals who find face-to-face interactions more challenging (Marriott and Buchanan, 2014). However, this control can also lead to discrepancies between online and offline identities, as people often present a polished version of themselves to gain social approval and positive feedback. Conversely, offline self-presentation benefits from real-time, synchronous communication, which can reduce misunderstandings and provide immediate feedback through nonverbal cues. This immediacy can foster more genuine interactions (Goffman, 1959). Additionally, extroverted individuals may find it easier to maintain consistent self-presentations across both online and offline contexts, while introverted individuals might use online platforms to explore and express aspects of their identity they find difficult to present face-to-face (Marriott and Buchanan, 2014; Valkenburg et al., 2006). Ultimately, both online and offline self-presentation have unique affordances and limitations, and the effectiveness of each can vary depending on the individual’s personality traits and the specific social context (Trepte and Reinecke, 2013). Understanding these differences is essential for exploring the complexities of identity construction and social interaction in the digital age.

### 1.2 Correlates of online-self-presentation

The dynamic interplay between the individual and their (online) social environment plays a crucial role both in personality expression and development (Neyer et al., 2014). Findings suggest that individuals employ distinct self-presentation strategies based on demographic characteristics and personality traits. Women are generally more inclined toward authentic self-presentation, sharing personal and emotional experiences to maintain social connections and seek support, while men are less likely to engage in authentic self-presentation and more likely to share content that emphasizes their achievements and status (Haferkamp et al., 2012). Both, men and women engage in idealized online self-presentation (Manago et al., 2008).

The Big Five personality traits (extraversion, neuroticism, agreeableness, openness, conscientiousness) exhibit correlations with self-presentation behaviors on Facebook, albeit studies occasionally yield inconsistent or even contradictory findings (Twomey and O'Reilly, 2017). Notably, findings pertaining to the neuroticism dimension consistently indicate a propensity for elevated neuroticism to be associated with inauthentic (false/idealized) self-presentation. This trend remains largely consistent across empirical investigations. The same inclination was observed regarding elevated scores in narcissism, and social anxiety as well as lower levels in self-esteem (Michikyan, 2020; Twomey and O'Reilly, 2017). Moreover, elevated levels of vulnerable narcissism and Machiavellianism have been linked to less congruent, or inauthentic, self-presentation on Instagram (Geary et al., 2021). Conversely, positive authentic (realistic) self-presentation tends to be more prevalent among users exhibiting high levels of self-esteem (Michikyan, 2020; Twomey and O'Reilly, 2017; Yang et al., 2017). Furthermore, a less stable self-concept is associated with presenting an idealized version of the self online (Fullwood et al., 2016; Michikyan, 2022; Strimbu and O'Connell, 2019; Yang et al., 2017). Moreover, individuals with low self-concept clarity exhibit a greater propensity to experiment with online identities and favor online self-presentation over face-to-face interactions (Fullwood et al., 2016; Strimbu and O'Connell, 2019). Individuals not only adapt their self-presentation to suit the online environment, but the environment also plays a crucial role in shaping personality development in a transactional manner (Neyer et al., 2014). This dynamic relationship underscores the profound impact of online contexts on the formation and expression of personality traits. Therefore, it is crucial to investigate how individuals differ in their face-to-face and online self-presentation.

The persistent nature of online content can cause users to curate their online personas excessively. Maintaining a discrepant online persona can lead to stress and feelings of inauthenticity, as individuals struggle to reconcile their real and virtual selves (Reinecke and Trepte, 2014). Over time, maintaining this discrepancy may result in identity confusion or dissatisfaction (Valkenburg et al., 2006).

The impostor phenomenon is closely related to identity discrepancies, particularly the gaps between one’s self-perception and the external image they present to others (Ibrahim et al., 2021). Individuals experiencing impostor phenomenon often feel a significant difference between how they view themselves and how they believe others perceive them (Clance and Imes, 1978). This creates a discrepancy between their internal identity and the persona they project to the outside world. This phenomenon can also influence the way individuals use social media and other online platforms. They might curate their online personas to emphasize successes and hide failures, further widening the gap between their public image and their private self-perceptions. This curated self-presentation can intensify feelings of impostor syndrome, as the praise and recognition they receive online may feel undeserved, reinforcing their sense of being an impostor (Leonhardt et al., 2017).

### 1.3 Assessing online self-presentation

Given the distinct nature of online and offline social environments, the question arises as to what extent people differ in terms of their self-presentations in each context. Therefore, Fullwood et al. (2016) developed a scale to measure the Presentation of Online Self (POSS). Based on theoretical assumptions and evidence from the literature, the authors developed 24 items addressing different domains in the context of online self-presentation. Exploratory factor analyses revealed a four-factor-structure based on 21 of the 24 items. A person’s online self-presentation behavior is captured on the factors (1) *Ideal Self* (nine items, e.g., “*I can show my best qualities online*“), which refers to the presentation of an idealized version of the self in online contexts, (2) M*ultiple Self* (five items, e.g., “*I enjoy acting out different identities online*“), which includes the presentation of different versions on different platforms, (3) *Consistent Self* (four items, e.g., “*I feel my personality online is the real me*“) describes the extent to which online and face-to-face self-presentation correspond and (4) P*reference for Online Self-Presentation* (three items, e.g., “*I find it easier to communicate in face to face contexts*“). Internal consistency of the scales ranges from *α* = 0.862 (ideal self) to *α* = 0.621 (consistent self). Due to the scope of the study, the initial version of the POSS is based on an adolescent sample. Adolescence is a central phase of identity development. Self-presentation plays a central role in the process of trying out different identities. This behavior plays a lesser role in adulthood. Therefore, Strimbu et al. (2021) investigated the factor structure of the POSS in an adult sample (POSSA). The Ideal Self factor was distributed among the other factors, resulting in the three factors (1) *Adaptable Self*, which refers to acting out different and more desirable online personas, (2) *Authentic Self*, comprising the discrepancy of the offline and online identity, and (3) *Freedom of Self Online*, describing the felt freedom to express oneself online.

Three subscales of the Impostor Profile demonstrate significant overlap with the POSSA factors, yet extend beyond the online context (Ibrahim et al., 2021). The subscale of O*ther-Self-Divergence* quantifies the degree to which individuals perceive environmental expectations as overstraining. *Alienation* encompasses the absence of a sense of authenticity and a high engagement in impression management. Additionally, the *Need for Sympathy* is conceptualized as an individual’s desire for popularity and goodwill from others. Accordingly, it is anticipated that individuals exhibiting high levels of other-self divergence, or alienation are less likely to present their authentic selves online. Instead, they tend to conform to environmental expectations in their online persona (Adaptable Self), and they may display a heightened preference for online self-presentation (Freedom of Self Online), owing to its greater controllability (Schlosser, 2020). Further, for individuals with a high need for sympathy employing adaptable selves in different online environments provides the opportunity to adapt to the expectations of the community to enhance popularity.

### 1.4 The present study

Online Identity refers to the personas and characteristics that individuals create and present on digital platforms. This identity can be an extension of the offline self or a significantly curated version tailored to meet social expectations or personal desires. The way individuals manage and perceive their online identities can have substantial implications for their mental health. Engaging with online communities allows individuals to connect with like-minded peers, providing emotional support, validation, and a sense of belonging. These social interactions are crucial for maintaining mental health and can significantly enhance life satisfaction (Ellison et al., 2007; Oh et al., 2014). Moreover, online platforms offer a unique environment for self-expression and identity exploration. Individuals can experiment with different aspects of their identity in a safe and relatively anonymous space, aiding in self-discovery and personal growth. This form of self-expression can bolster self-esteem and foster a more robust sense of self (Bargh and McKenna, 2004). On the other hand, maintaining a highly curated or idealized online persona can lead to stress and feelings of inauthenticity, contributing to mental health issues such as anxiety and low self-esteem (Gonzales and Hancock, 2011; Valkenburg et al., 2006). Moreover, discrepancies between one’s online and offline identities can exacerbate feelings of fraudulence and impostor syndrome (Leonhardt et al., 2017). Considering that SNSs and, therefore, online identity has become an integral part of interpersonal interaction and have numerous implications for well-being, mental health and socialization, a valid instrument for recording online self-presentation is of great relevance. To the best of our knowledge to date no German version of the POSSA is available. Therefore, in this study, we investigate the structure and construct validity of the German version of the POSSA, referred to as the DE-POSSA.

## 2 Methods

### 2.1 Participants and procedure

Participants were recruited for the online survey via email and various social media platforms. Additionally, students from a Northern German University were incentivized with achievement points for their participation. The survey garnered a total of 541 responses. However, five cases had to be excluded as the participants were under 18 years old, resulting in a final sample size of 536 participants. With this sample size, the study exhibits a robust power of 0.98 to detect effect sizes of ρ = 0.20.

Regarding demographics, the participant composition was as follows: 46,27% identified as female, 53,17% as male, and 0,56% as non-gender specific. The age of participants ranged from 18 to 76 years, with a mean age of *M* = 26.73 (SD = 8.41).

The research data have been securely archived and are accessible via the Open Science Framework link: https://osf.io/kew8b/?view_only=72aaa941f445411d815e4001b41a9da8.

### 2.2 Measures

#### 2.2.1 DE-POSSA

The Presentation of Online Self Scale for Adults (POSSA; Strimbu et al., 2021) is a self-report instrument comprising 17 items, delineating three factors: Adaptable Self (reflecting experimentation with various online identities; *ɑ* = 0.87), Authentic Self (indicating congruence between true and online self; *ɑ* = 0.72), and Freedom of Self Online (capturing the preference for online over face-to-face self-presentation; *ɑ* = 0.77) in English language. Respondents rate items on a 5-point Likert scale ranging from 1 (strongly disagree) to 5 (strongly agree). The translation process involved two primary steps. Initially, one author and a bilingual expert in the research field independently translated the 21 items of the POSSA into German. The translations were then compared, discussed, and adjusted to create a consensus version. Next, this German version (DE-POSSA) was translated back into English by a bilingual professional and an English native speaker fluent in German, both without expertise in the research field. The back-translations showed high congruence with the original SAAM in terms of wording and semantic content. In a subsequent step, a small sample (*N* = 5) completed the preliminary DE-POSSA and provided feedback on comprehension. Only minor adjustments were needed to enhance clarity while maintaining the intended structure. The final version of the DE-POSSA was administered in the survey.

#### 2.2.2 Rosenberg self-esteem scale

The Rosenberg Self-Esteem Scale (RSES; Rosenberg, 1965), as adapted by Von Collani Herzberg (2003), comprises 10 items in German language and demonstrates robust reliability (*ɑ* = 0.84), assessing two factors: Self-Diminishing and Positive Self-Esteem. Participant’s rate items on a 4-point Likert scale ranging from 1 (not true at all) to 4 (is entirely true).

#### 2.2.3 Impostor-profile

The Impostor-Profile 30 (IPP; Ibrahim et al., 2021) encompasses six subscales alongside the IPP total score. Comprising a total of 30 items, such as "Despite former success, I have a strong fear of failure," the IPP measures the expression of impostor phenomenon (IP) on a 10-point Likert scale ranging from 1 (Not like me at all) to 10 (Very much like me). Reliability estimates range from good to excellent (*ɑ* = 0.94–0.72; Ibrahim et al., 2022), with the Need for Sympathy scale demonstrating lower reliability (*ɑ* = 0.67; Ibrahim et al., 2021). The original scale by Ibrahim et al. (2021) was conceptualized in German. This German version was presented to the participants.

#### 2.2.4 Social network site use

For evaluating SNS usage, participants were queried regarding their usage of Instagram (Do you have an Instagram account?). Additionally, participants reported their count of followers on Instagram (How many followers do you have on Instagram?). A higher number of followers can be perceived as a form of social validation, suggesting that the individual is popular or influential. This perception can influence how users present themselves online, striving for a positive image that attracts and retains followers (Utz et al., 2012).

### 2.3 Design and data analysis

This research utilized a cross-sectional design through an online survey platform.

First, we tested the fit of the three-factor model to data of a German sample. Convergent and discriminant validity was computed in a single SEM model with correlated latent variables. For convergent validity, the subscales of the DE-POSSA with those of the IPP (Ibrahim et al., 2021) were correlated. Discriminant validity was determined by correlating the DE-POSSA with the German version of the Rosenberg Self-Esteem Scale (Von Collani and Herzberg, 2003).

**Figure 1:**
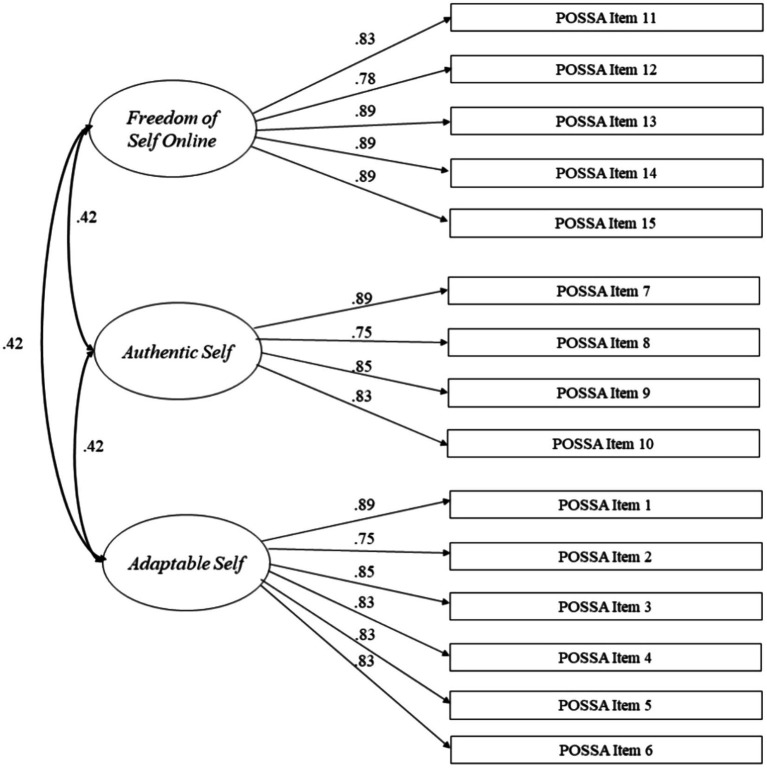
Structural equation model of the three-factor-structure of the DE-POSSA. Item numbers according to the original POSSA by Strimbu et al. (2021).

The study employed confirmatory factor analysis to examine the proposed three-factor model of the DE-POSSA (Figure 1). The latent variables (Adaptable Self, Authentic Self, Freedom of Self Online) were operationalized with four to seven items. Error terms within the model were uncorrelated, while correlations between factors were freely estimated. The assumption of multivariate normality was violated (Mardia’s normalized estimate of multivariate kurtosis = 71.63). Consequently, the R-package *lavaan* (Rosseel, 2012) was employed, utilizing the robust MLM estimator, which implements maximum likelihood estimation with robust standard errors and a Satorra-Bentler scaled test statistic to adjust for non-normality. Latent associations were evaluated employing the structural equation modeling (SEM) framework.

To evaluate convergent validity, the Pearson Product Moment Correlation was employed, involving three subscales of the Impostor Profile, i.e., *Other-Self-Divergence*, *Alienation*, and *Need for Sympathy*.

To examine incremental validity, we tested whether the POSSA factors predicted the number of followers on Instagram beyond self-worth. Instagram is one of the most popular SNSs (We are Social, DataReportal, Meltwater, 2024) and plays a special role for self-presentation due to its inherent features, such as pictures and stories (Geary et al., 2021). Higher self-esteem is positively correlated with both increased authenticity in online self-presentations and a larger follower count (Grieve et al., 2020; Serra and Campaniço, 2024). This relationship appears plausible, given that profiles aligning with community expectations while simultaneously conveying authenticity might attract more followers as it is perceived more positively (Schlenker and Wowra, 2003; Yau and Reich, 2019). It can be assumed that this correlation also persists beyond the influence of self-esteem, as a strategically good online self-presentation (which consequently shows a certain discrepancy to face-to-faceself-presentation) attracts more followers.

## 3 Results

### 3.1 Model fit

To address the ordinal data and the deviations from normal distribution, we used maximum likelihood estimation with robust standard errors and a Satorra-Bentler scaled test statistic. We compared the three-factor model by Strimbu et al. (2021) with a one-factor solution. The three-factor model displayed a reasonable fit to the data. The *χ*^2^/df ratio is 2.96 which is acceptable. According to Hair et al. (2019) a Comparative Fit Index values of 0.90 or higher are considered acceptable, a Tucker-Lewis Index value above 0.90, Root Mean Square Error of Approximation values between 0.05 and 0.08 represent a reasonable fit, and a Standardized Root Mean Square Residual value of 0.08 or lower is considered a good fit. Thus, the Comparative Fit Index (CFI = 0.919), the Tucker-Lewis Index (TLI = 0.902), the Root Mean Square Error of Approximation (RMSEA = 0.075 with 90% CI = 0.065, 0.086), and the Standardized Root Mean Square Residual (SRMR = 0.070) indicated a reasonable to good fit of the model to the data close to the original scale (Strimbu et al., 2021). The one-factor solution was not acceptable [*χ*^2^ (105) = 2398.839, *p* < 0.001] with CFI (0.805), the Tucker-Lewis Index (0.773), the RMSEA (0.115 with 90% CI = 0.104, 0.126), and the SRMR (0.091). Akaike information criterion (20091.526 vs. 20486.544) and Bayesian information criterion values (20232.903 vs. 20615.068) indicated an improvement of the one- versus three-factor solution.

### 3.2 Descriptive analyses

Table 1 provides an overview of the descriptive analyses of the DE-POSSA subscales as well as gender differences. Given the significantly limited sample size of the ‘divers’ category, which comprises only three cases, we have decided to exclude this group from the current analysis to avoid issues with statistical power and the reliability of the results analogous to the decision by Strimbu et al. (2021). Gender effects for the DE-POSSA score were found for the Adaptable Self factor (Hedges’ *g* = 0.21). In line with findings of the original POSSA, men scored significantly higher than women. No differences between genders were found for authentic self (*g* = −0.054) and Freedom of Self Online (*g* = 0.11). Higher age predicted lower scores on the factors Adaptable Self (*β* = −0.10; *p* = 0.024) and on the Freedom of Self Online (*β* = −0.16, *p* < 0.001).

**Table 1:** Gender differences and descriptive analyses of the presentation of online self adult (POSSA) factors.

POSSA factor	*M* (SD)			Hedges’ *g*	Skewness	Kurtosis
	Total *N* = 533	Female *N* = 248	Male *N* = 285			
Adaptable self	1.42	1.35	1.49	0.21	2.29	5.20
	(0.70)	(0.60)	(0.78)			
Authentic self	4.10	4.13	4.08	−0.05	−0.80	−0.14
	(0.83)	(0.84)	(0.81)			
FOS online	1.91	1.87	1.95	0.11	0.83	0.21
	(0.72)	(0.71)	(0.72)			

*N = 533; FOS, Freedom of self.*

Further, factor correlations matched those found by Strimbu et al. (2021) with a significant negative correlations of Authentic Self with Adaptable Self (*r* = −0.53, *p* < 0.01) and Freedom of Self Online (*r* = −36, *p* < 0.01) and a significant positive correlation of Adaptable Self and Freedom of Self Online (*r* = 0.48, *p* < 0.01).

### 3.3 Reliability

Internal consistency was assessed with Cronbach’s α values. Reliabilities for Adaptable Self (0.88), Authentic Self (0.76), and Freedom of Self Online (0.75) were satisfactory and close to those found for the original scale (Strimbu et al., 2021).

### 3.4 Convergent validity

To assess convergent validity the three factors of the DE-POSSA were correlated with three subscales of the IPP (Ibrahim et al., 2021) which capture aspects related to self-presentation (Table 2). In line with assumptions the POSSA-factors Adaptable Self and Freedom of Self Online showed significant positive correlations with Alienation and Other-Self-Divergence, indicating a less consistent online vs. face-to-faceself-presentation for individuals who generally feel less authentic and a high pressure to match the expectations of others. This pattern was supported by negative correlations of the DE-POSSA-factor Authentic Self with IPP Alienation and Other-Self-Divergence. Lastly, consistent with assumptions, a significant positive association emerged between IPP Need for Sympathy and Adaptable Self, implying that individuals who have a strong desire for popularity adapt their online self-presentation to match the expectations of different online communities.

**Table 2:** Presentation of online self adult (POSSA) factor correlations with impostor profile (IPP) subscales.

IPP Subscale	POSSA adaptable Self	POSSA authentic self	POSSA FOS online
Other-self-divergence	0.23^**^	−0.26^**^	0.24^**^
Alienation	0.37^**^	−0.36^**^	0.38^**^
Need for sympathy	0.09^*^	0.05	0.07

FOS, Freedom of self; OS, Other-self. ^*^p < 0.05; ^**^p < 0.01.

### 3.5 Incremental validity

To assess incremental validity, it was tested whether the DE-POSSA factor scores could predict the number of followers on Instagram over and beyond self-esteem measures. For this purpose, a hierarchical regression analysis was used. 438 participants indicated to use Instagram and were included in the analyses. The results are displayed in Table 3. Two baseline models (Model 1: DE-POSSA, Model 2: RSES) were calculated to assess the amount of variance accounted for by the DE-POSSA factors and the RSES subscales as the only predictor. The DE-POSSA factors accounted for more variance in the number of followers on Instagram (*R^2^* = 0.03, *p* = 0.006) than RSES subscales (*R^2^* = 0.01, *p* = 0.111). A significant positive association emerged for Adaptable Self on the number of Instagram followers while the factor Freedom of Self displayed a significant negative correlation. To test the incremental value of the DE-POSSA against age, gender, and the RSES, a hierarchical regression was calculated in Model 3. First, age and gender were entered, followed by the RSES subscales in a second step, and the POSSA factors in the last step. Adding the DE-POSSA led to a significant increase in the amount of variance explained for followers on Instagram (∆*R^2^* = 0.03, *p* = 0.005). Again, Adaptable Self and Freedom of Self Online contributed to a significant increase in R^2^.

**Table 3:** Regression Analyses models to assess incremental validity of the POSS over RSES positive self-esteem and self-diminishing for number of followers on instagram.

Predictor	Satisfaction with life (SWLS)		
	Model 1	Model 2	Model 3
1. Gender			−0.07
2. Age			0.03
3. RSES positive self-esteem	0.16^*^		0.18^*^
4. RSES self-diminishing	0.10		0.12
5. POSSA adaptable self		0.16^**^	0.15^*^
6. POSSA authentic self		−0.05	−0.06
7. POSSA freedom of self online		−0.12^*^	−0.13^*^
∆*R*^2^			0.03^**^
Model *R*^2^	0.01	0.03^**^	0.05^**^

*N* = 438, entries are standardized β-coefficients of the final model. ^*^p < 0.05; ^**^p < 0.01.

## 4 Discussion

Self-presentation is an integral part of interpersonal interaction, manifesting both consciously and unconsciously. Individuals curate aspects of their persona in response to environmental demands and inherent personality traits, potentially giving rise to authenticity discrepancies. SNSs have elevated the scope of self-presentation, allowing for enhanced control and selective information disclosure compared to offline interactions. This advancement prompts an investigation into the variance between online and face-to-face self-presentation. Initially, the Presentation of Online Self Scale (POSS) was developed to assess this phenomenon among adolescents. Subsequently, Strimbu et al. (2021) extended the applicability of the scale to an adult population (POSSA). The original scale was administered in English and assessed using a sample of English-speaking participants, predominantly comprising US and Irish citizens (70.5%). Given the global ubiquity and cultural diversity of SNS usage, it is imperative to validate such scales across different cultural and linguistic contexts. Accordingly, this study explores the efficacy of the German adaptation of the POSSA, the DE-POSSA.

Gender differences were only found for the Adaptable Self factor but not the Freedom of Self factor, analogous to Strimbu et al. (2021). Further, no gender differences were found for authentic self. However, in the original article, the found difference in means for Authentic Self displayed a small magnitude.

The three-factor structure of the POSSA was affirmed in the German version, denoted as DE-POSSA. This adaptation demonstrated robust model fit indices, which align closely with those reported in the original study by Strimbu et al. (2021). Additionally, the internal consistency of the scales was evaluated and found to be satisfactory. A significant negative correlation was found for Authentic Self with both Adaptable Self and Freedom of Self Online, while Adaptable Self and Freedom of Self Online were significantly positively related. These patterns also emerged in the original study.

Moreover, convergent and incremental validity of the DE-POSSA were assessed. As assumed, the DE-POSSA factors showed strong correlations with subscales of the Impostor Profile, that refer to discrepancies between the private and the public self (Ibrahim et al., 2021). The subscales Alienation and Other-Self-Divergence were associated with more discrepancies between online and face-to-face self-presentation, that is with higher scores on the Adaptable Self and Freedom of Self Online factor. Individuals experiencing a perceived lack of authenticity or believe that they are unable to meet the high expectations set by others, may utilize online self-presentation as a strategy to uphold their desired image. This approach likely facilitates the fulfillment of such expectations in an online environment than offline. The enhanced control over self-disclosed information in digital contexts provides individuals with the means to selectively portray themselves in a manner that aligns with these expectations (Bareket-Bojmel et al., 2016; Yau and Reich, 2019). This capacity to manage impressions extensively may reduce discrepancies between actual self-perceptions and the expectations of others, thus serving as a compensatory mechanism for those feeling inauthentic. Additionally, the elements of anonymity and asynchrony inherent in digital communication provide a safer space for these individuals, who may have persistent fears of being unmasked as frauds (Ibrahim et al., 2021). These online characteristics enable them to reveal aspects of their true selves without the immediate risk of compromising their carefully curated personas. This dynamic is substantiated by the observed negative correlation between the two subscales of the IPP and the Authentic Self factor. Specifically, individuals who experience feelings of inauthenticity or fears regarding the high expectations of others are less likely to present themselves authentically online. These findings indicate that the lack of perceived authenticity in personal identity may extend into digital interactions, where such individuals continue to manage their self-presentation strategically rather than expressing their true selves. This pattern highlights the potential for online environments to both mask and perpetuate underlying struggles with authenticity and self-concept (Fullwood et al., 2016; Strimbu and O'Connell, 2019). Lastly, the subscale Need for Sympathy is only associated with Adaptable Self. Individuals who seek popularity and the goodwill of others are more able to conform to the norms and expectations of various online communities than in offline settings. These communities likely serve as a valuable source of positive feedback, which is easier to manage than in the more complex real world, which includes non-verbal cues, immediate feedback and direct consequences, as well as navigating diverse social roles and contexts simultaneously in changing environments and situationally factors (Bareket-Bojmel et al., 2016).

Incremental validity was tested using hierarchical regression. The results showed that DE-POSSA factor scores predict the number of followers on Instagram over and beyond positive self-esteem and self-diminishing as assessed by the German version of the Rosenberg Self-Esteem Scale (Von Collani and Herzberg, 2003).

Adaptable Self and Freedom of Self scores, but not Authentic Self scores, have significantly contributed to predicting the number of Instagram followers. A higher score on the Adaptable Self factor was associated with a higher follower count, while the opposite was found for Freedom of Self. This finding supports the assumption that individuals who value positive feedback from others present themselves online in a manner that appeals to specific communities, thereby generating and desiring a larger follower count. This aligns with findings that self-esteem is associated with follower count, and that likes and followers can boost self-esteem (Grieve et al., 2020; Serra and Campaniço, 2024). Individuals who can better express their true selves online likely also selectively curate their followers and may benefit from control over visibility and the anonymity provided by online platforms.

### 4.1 Limitations

A limitation arises from the composition of the sample. Due to the notably small sample size of the diverse category, consisting of only three cases, we have opted to exclude this group from the current analysis to ensure statistical power and result reliability. Since there was a gender difference between men and women in the Adaptable Self factor, it is important to recognize the need for further data collection to adequately address the group of diverse individuals in future studies. Furthermore, while we have assessed the convergent validity, we have not evaluated the discriminant validity of the DE-POSSA against measures of self-presentation in offline contexts. Therefore, it remains unclear to what extent the DE-POSSA distinctly measures online self-presentation as opposed to general self-presentation behaviors. Additionally, we have only considered follower counts on Instagram as a criterion for incremental validity. However, it would be of great interest to determine whether this relationship also holds with followers or friends on other social networking sites (SNSs). Moreover, the number of followers is a very general indicator of SNS usage. More specific aspects of usage, both cross-platform and platform-specific, such as the number of reactions to posts (comments, likes), the type of information published, or the use of tools that explicitly alter self-presentation (e.g., filters), are more closely related to self-presentation but are also more challenging to measure (Stöven and Herzberg, 2021).

### 4.2 Conclusion

In summary, the psychometric properties indicate that the DE-POSSA is a reliable tool for assessing self-presentation behaviors in a German-speaking context, thereby supporting its utility for cross-cultural research in the domain of online interpersonal interactions.

Further, the findings indicate that online platforms provide an alternative environment for individuals to shape and refine their self-presentation in ways that are specifically designed to gain approval and positive reinforcement or express facets of the self, which feel inappropriate in the offline world. In the scope of identity and self-concept development (Neyer et al., 2014; Yang et al., 2017), this strategic alignment with community norms not only enhances users’ ability to receive the desired social rewards, but might also reinforce the behavior, potentially influencing users’ self-perception and identity over time. Applying the POSSA might contribute to bringing further insights into how online self-presentation might shape the development of self-concept and identity in future research.

## Data Availability

The datasets presented in this study can be found in online repositories. The names of the repository/repositories and accession number(s) can be found at: https://osf.io/kew8b/?view_only=72aaa941f445411d815e4001b41a9da8.
